# Electrical stimulation of the lower eyelid orbicularis oculi muscle improves periocular dark circles

**DOI:** 10.1111/srt.13678

**Published:** 2024-04-14

**Authors:** Yukiko Yasui, Hiroshi Kato, Shuntaro Ogura, Masayo Kimura, Aki Kato, Yoshio Hirano, Hiroshi Morita, Tsutomu Yasukawa, Ako Kurachi, Soshi Takeda, Akimichi Morita

**Affiliations:** ^1^ Department of Geriatric and Environmental Dermatology Nagoya City University Graduate School of Medical Sciences Nagoya Japan; ^2^ Department of Ophthalmology and Visual Science Nagoya City University Graduate School of Medical Sciences Nagoya Japan; ^3^ MTG Co. Ltd Nagoya Aichi Japan

**Keywords:** clinical dark circle score, electrical muscle stimulation, spectrophotometry, TcPO2

## Abstract

**Background:**

We developed and tested the safety and efficacy of a cosmetic device to improve dark circles using electrical muscle stimulation of the orbicularis oculi muscle.

**Methods:**

Overall, 18 participants (36 eyes) were studied. The following five items were evaluated before and after the intervention:(1) the Clinical Dark Circle Score using clinical findings and photographs, (2) transcutaneous oxygen partial pressure (TcPO_2_) on the lower eyelid, (3) thermography, (4) two‐dimensional laser blood flowmetry, and (5) spectrophotometry.

**Results:**

The mean score at baseline was 2.0 ± 0.90 (mean ± standard deviation), and that at the end of the study was 1.2 ± 1.0 (Wilcoxon signed‐rank sum test, *p* < 0.0001), indicating a significant reduction. The spectrophotometer showed a significant decrease in a* and L* values before and after use (Wilcoxon signed‐rank sum test, *p* < 0.0001). There was also a weak negative correlation between the change in score and the change in blood flow and TcPO_2_ measured using a laser perfusion device (Spearman's rank correlation coefficient, *r* = –0.32 and –0.39, respectively). Stratified analysis of the baseline score showed a strong negative correlation between the change in score and the change in spectrophotometric a* in the subjects/group with mild periocular dark circles (Spearman's rank correlation coefficient, *r* = –0.46). Contrastingly, no correlation was observed for any of the measurements in the subjects/group with severe periocular dark circles. After 1 month, no device‐related ophthalmic adverse events were observed in any of the participants.

**Conclusion:**

Electrical muscle stimulation could improve periocular dark circles, especially in the subjects/group with mild periocular dark circles, and was safe.

## 1 INTRODUCTION

Periocular dark circles make a person appear older or fatigued and are a major cosmetic problem.^1^ They are caused by several factors, including facial anatomy, soft tissue problems such as those involving the orbicularis oculi muscle, and pigmentation of the skin.^2^

### 1.1 Anatomical problems

Due to aging and other factors, the orbital septum relaxes and the internal orbital fat tissue protrudes. This is particularly noticeable at the lower edge of the orbit owing to the downward pull of gravity.^3^ The protrusion itself is not a cosmetic problem, however, the shadowing caused by this protrusion leads to the accentuation of dark circles around the eyes.^4^ This condition is more noticeable when the skin and subcutaneous tissue are thinner.

### 1.2 Soft tissue problems

The skin of the lower eyelids is one of the thinnest skin layers of the entire body, and it often directly reflects the color tone of the dermis, orbicularis oculi muscle, and other tissues directly beneath it.^5^ The lower eyelids are anatomically prone to fluid retention due to edema, which is known to be exacerbated in the morning after a high‐salt diet or sleep deprivation and often takes on a purplish hue.^6^

### 1.3 Pigmentation

The pigmented appearance may be caused by an excess of hemoglobin breakdown products, such as hemosiderin and biliverdin, due to subcutaneous tissue edema, or by pigmentation of the skin or subcutaneous tissue, such as melasma, dermal melanocytosis, or post‐inflammatory hyperpigmentation.^7^

**FIGURE 1:**
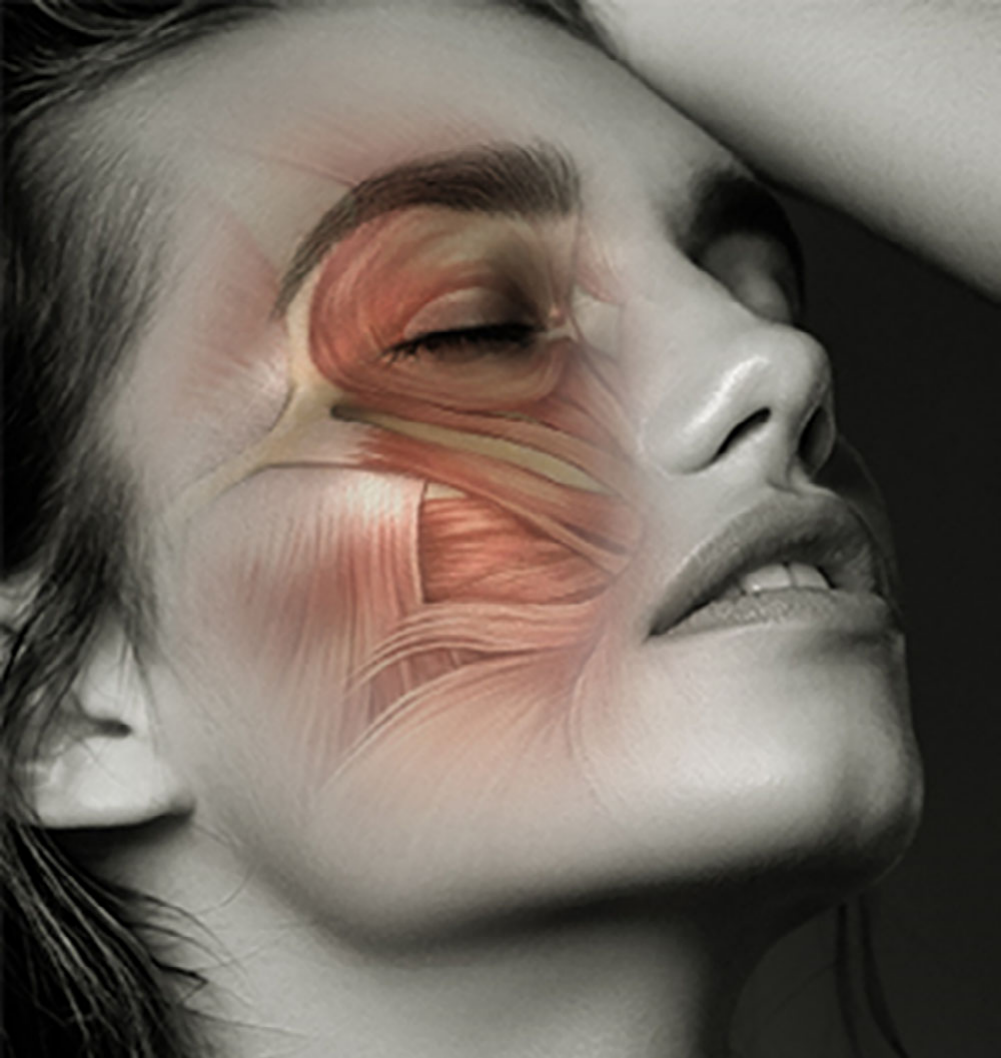
Schematic of facial muscles. The face is a complex arrangement of facial muscles. The orbicularis oculi muscle, located just below the eyelid, is the muscle responsible for the movement of the surrounding area and the formation of wrinkles.

Several treatments have been proposed for periocular dark circles, including surgery, filler injections, topical hydroquinone, and laser therapy.^8^ However, most of these treatments are invasive and require a visit to a medical facility. The orbicularis oculi muscle is a major soft tissue component involved in periocular circles (Figure 1). Advanced surgical procedures targeting the orbicularis oculi muscle^9^ have been reported in recent years, but very few conservative treatments target the orbicularis oculi muscle. In this study, we developed an electrical muscle stimulation (EMS) device to stimulate the facial nerve branch that enters the orbicularis oculi muscle, consequently stimulating the lower eyelid. This study also aimed to verify the safety and efficacy of this device.

## 2 MATERIALS AND METHODS

### 2.1 Test product

An EMS device (MTG Co., Ltd., Nagoya, Japan) was manufactured according to the specifications in Supplementary File 1, with an output of 1 mA (frequency, 20 Hz; pulse shape, square wave; pulse duration, 50 μs). The orbicularis oculi muscle is divided into three parts: the orbital part, the palpebral part, and the lacrimal part, and this product electrically stimulates the zygomatic branch of the facial nerve, activating all the orbicularis oculi muscles on the lower side of the eyelid. The transcutaneous partial pressure of oxygen (TcPO_2_) was measured using a PERIFLUX 6000 (Integral Corporation, Tokyo, Japan). The probe was placed on the participant's lower eyelid and the participant was allowed to rest for 15 min before measurements were taken according to the prescribed protocol. Thermography was performed with InfReC R450 (Nippon Avionics, Yokohama, Japan), and measurements were taken after the participant rested for 20 min in a room with the room temperature set at 26°C. A CM‐25cG spectrophotometer (Konica Minolta Japan, Inc., Tokyo, Japan) was used to measure the color tone of the lower eyelid. Laser blood flow measurements were performed using OMEGAZONE OZ‐2 (OmegaWave, Inc., Tokyo, Japan). TcPO_2_ measurements were performed after all other examinations were completed because the probe had to be placed on the lower eyelid, which might interfere with other measurements.

### 2.2 Study design

**FIGURE 2:**
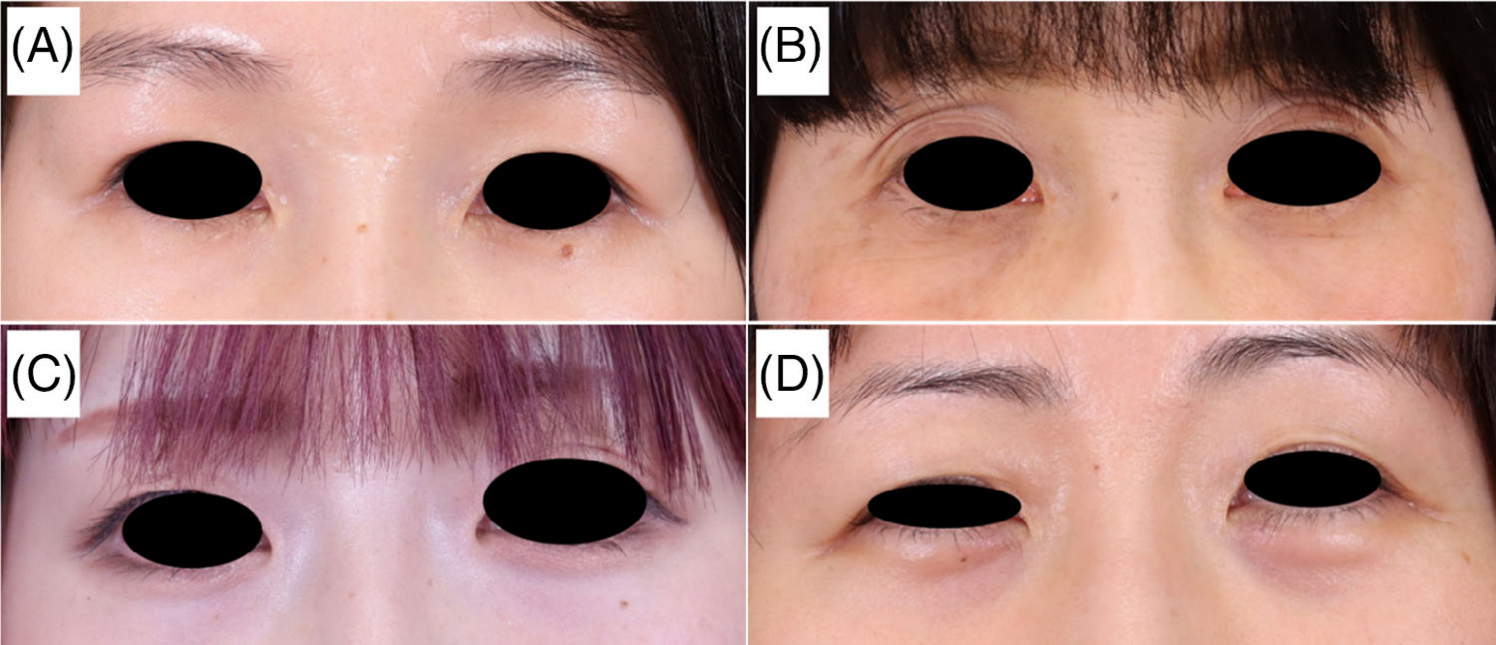
Periocular dark circles graded by two dermatologists. (A) CDC score 0, (B) CDC score 1, (C) CDC score 2, (D) CDC score 3, CDC score: clinical dark circle score.

The following ophthalmologic screening examinations were performed before the study^1^: presence of eye pain^2^; distance of corneal reflex^3^; ocular movements^4^; visual acuity^5^; intraocular pressure^6^; anterior segment examination by slit‐lamp microscopy^7^; fundus examination using an inverted ophthalmoscope, ultra‐widefield retinal imaging, and optical coherence tomography with mydriasis; and^8^ visual field measurement by Humphrey visual field analyzer. Initial evaluations included^1^ the Clinical Dark Circle (CDC) Score (assessed by two dermatologists [H.K., Y.Y.]) and scored as dark, slightly dark, light, and almost no dark circles from clinical findings and photographs (Figure 2A–D),^2^ lower lid TcPO_2_,^3^ thermography,^4^ two‐dimensional laser blood flow measurements, and^5^ spectrophotometry. After the initial examinations, the participants used the device on their bilateral lower eyelids once a day for 3 min for 1 month, and the same tests were repeated after 1 month to assess safety of the device and improvement of periocular dark circles before and after the intervention.

### 2.3 Study participants

Twenty participants were enrolled in the study. The inclusion criteria were: participants aged ≥20 years and aware of their periocular dark circles. In addition, the following exclusion criteria were defined: ptosis, eye movement disorder, corneal disease, uveitis, chorioretinal disease, glaucoma, history of internal or external eye surgery, history of epilepsy or seizures, significant abnormalities in the skin of the study area, and presence of a metallic medical device such as a pacemaker or clip in the body. Two subjects were excluded due to eye disease and the final number of subjects was 18 and individual eye included as a discrete value in clinical scoring and other measurements. The subjects were 6 men and 12 women with a mean age of 41.3 (25–57) years. CDC scores were also classified into mild periocular dark circles group (CDC score 0 or 1) and severe periocular dark circles group (CDC score 2 or 3), and each subjects were 6 and 12.

### 2.4 Statistical analysis

The Wilcoxon signed‐rank sum test was used to evaluate the correlation between the degree of improvement in the CDC score and measurements of^1^ TcPO_2_,^2^ thermography,^3^ two‐dimensional laser blood flowmetry, and^4^ spectrophotometry. Spearman's correlation coefficients were calculated for the change in CDC scores and changes in the parameters. CDC scores were also classified into mild periocular dark circles group and severe periocular dark circles group, and Spearman's correlation coefficients were calculated for the change in the CDC score and the change in the parameters. All statistical analyses were performed using GraphPad Prism software (MDF Co., Ltd., Tokyo, Japan).

## 3 RESULTS

**FIGURE 3:**
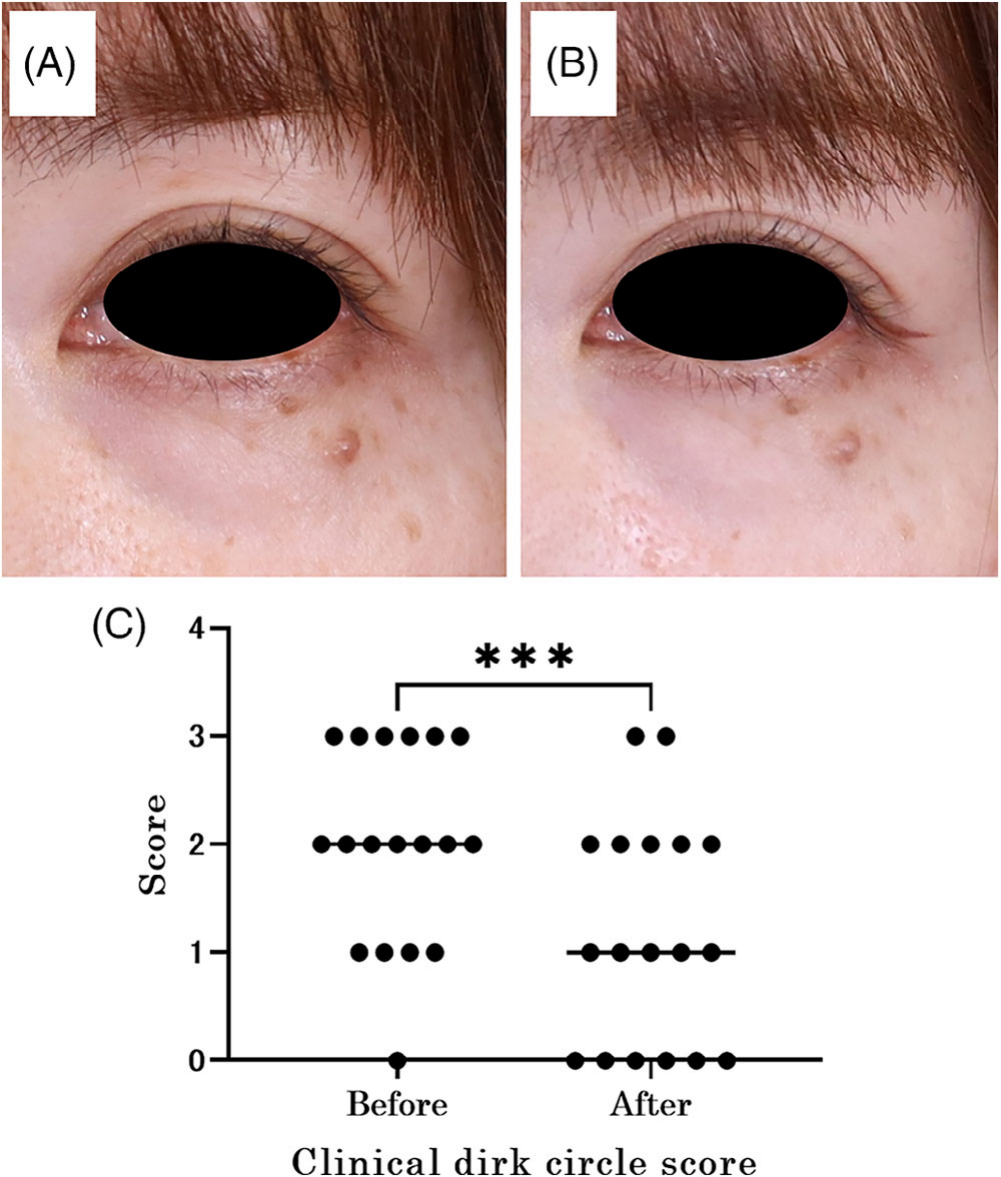
Representative cases and changes in CDC score before and after device use. (A) Photograph of a representative case at baseline with a CDC score of 3, (B) Photograph of the same patient at the end of the study. The CDC score has improved to 2. (C) Change in score from baseline to the end of the study. Significant decrease in the CDC score (Wilcoxon Signed Rank Sum Test, *p* < 0.0001). CDC score: clinical dark circle score.

**FIGURE 4:**
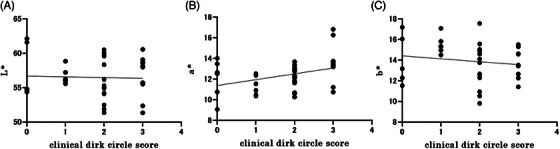
Correlation between CDC score at baseline and spectrophotometric value. (A) Correlation between L* and CDC scores at baseline. No significant correlations are observed. (B) Correlation between a* values and CDC scores at baseline. Weak positive correlations. (Spearman's rank correlation coefficient *r* = 0.358, *p* = 0.032). (C) Correlation between b* values and CDC scores at baseline. No significant correlations are observed. CDC score: clinical dark circle score.

**FIGURE 5:**
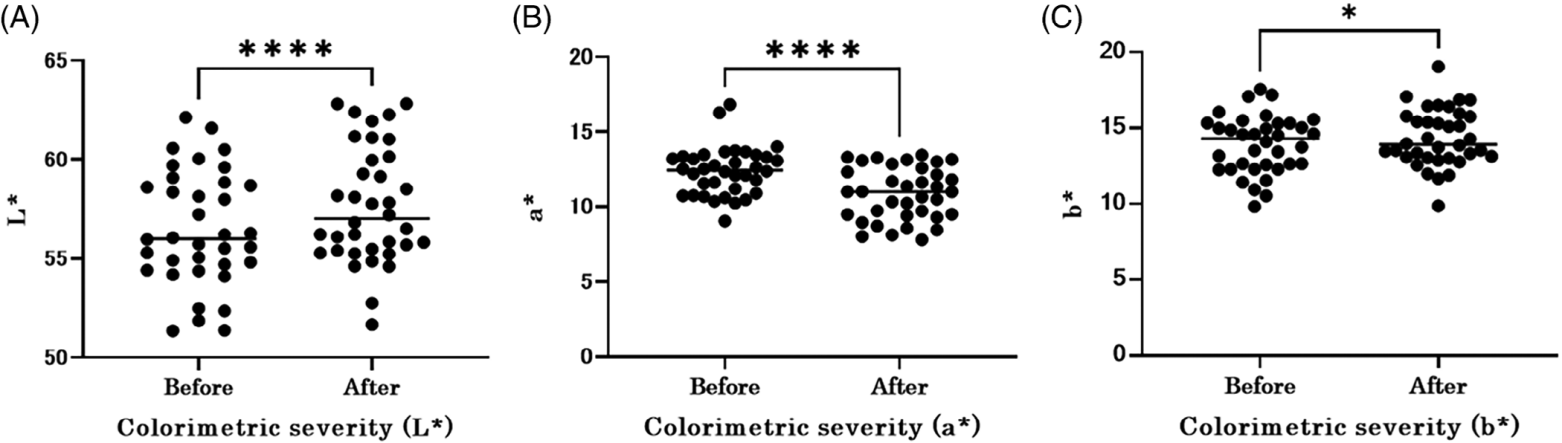
Change in spectrophotometric values before and after the test. (A) L* values increase at the end of the study period, which can be interpreted as an increase in luminance. (Wilcoxon signed‐rank sum test, *p* < 0.0001). (B) a* values decrease at the end of the study period. a* indicates skin pigmentation, which can be interpreted as redness (Wilcoxon signed‐rank sum test, *p* < 0.0001). (C)ｂ* values do not change at the end of the period studied, which can be interpreted as no changes in yellowness.

**FIGURE 6:**
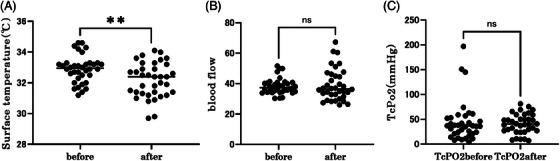
(A) Change in skin surface temperature before and after the test. Skin surface temperature decreased significantly (Wilcoxon signed rank sum test, *p* < 0.01). (B) Changes in blood flow before and after the test. No changes in blood flow are observed. (C) Change in lower lid TcPO2 before and after the test. No changes in TcPO2 are observed. TcPO2: transcutaneous partial pressure of oxygen.

The study started with a total of 36 eyes from 18 participants. Two participants, one with a retinal tear and the other with suspected glaucoma on visual field testing, were excluded from the study. The mean CDC score at baseline was 2.0 ± 0.90 (mean ± standard deviation), and the mean CDC score at the end of the study was 1.2 ± 1.0 (Wilcoxon signed‐rank sum test, *p* < 0.0001) (Figure 3A–C), indicating a significant reduction. In addition, a weak positive correlation was found between a*, which indicates redness, and the initial CDC score (Figure 4A–C) (Spearman's rank correlation coefficient, *r* = 0.36, *p* = 0.032). In addition, the a* values decreased significantly after use (Wilcoxon signed‐rank sum test, *p* < 0.0001). Brightness (L*) also increased significantly after device usage (Wilcoxon signed‐rank sum test, *p* < 0.0001) (Figure 5A–C). The surface temperature measured by thermography showed a significant decrease after device use (Wilcoxon signed‐rank sum test, *p* < 0.01), whereas TcPO_2_ and laser blood flowmetry showed no significant changes (Figure 6A–C).

**FIGURE 7:**
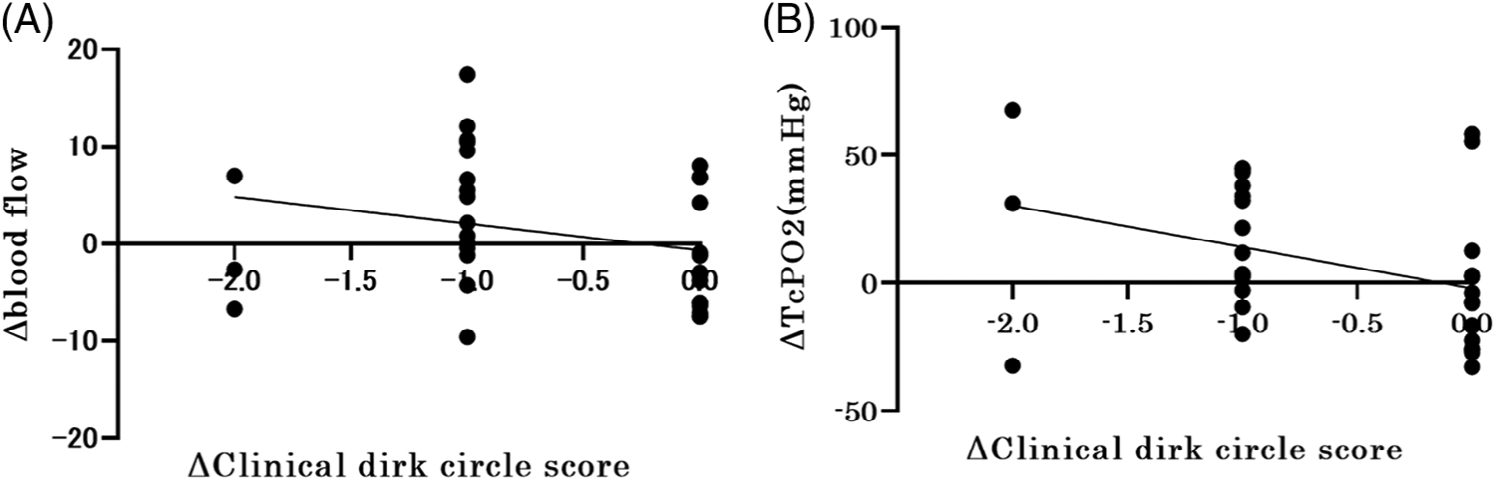
(A) Correlation between change in CDC score and blood flow values. (B) Correlation between change in CDC score and lower lid TcPO2. An improvement in the score is associated with a greater increase in TcPO2 (Spearman's rank correlation coefficient, *r* = −0.39, *p* = 0.027). CDC score: clinical dark circle score; TcPO2: transcutaneous partial pressure of oxygen.

**FIGURE 8:**
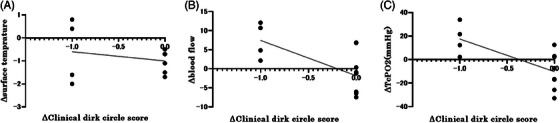
Correlation between change in score and change in parameters in the subjects/group with mild periocular dark circles (CDC score 0−1 at baseline). (A) Correlation with change in surface temperature. (B) Correlation with changes in blood flow. Patients with lower CDC scores have higher blood flow values (Spearman's rank correlation coefficient, *r* = −0.615, *p* = 0.049). (C) Correlation with lower lid TcPO2 changes. The subjects/group with mild periocular dark circles values have higher TcPO2 (Spearman's rank correlation coefficient, *r* = −0.615, *p* = 0.049). CDC score: clinical dark circle score; TcPO2: transcutaneous partial pressure of oxygen.

**FIGURE 9:**
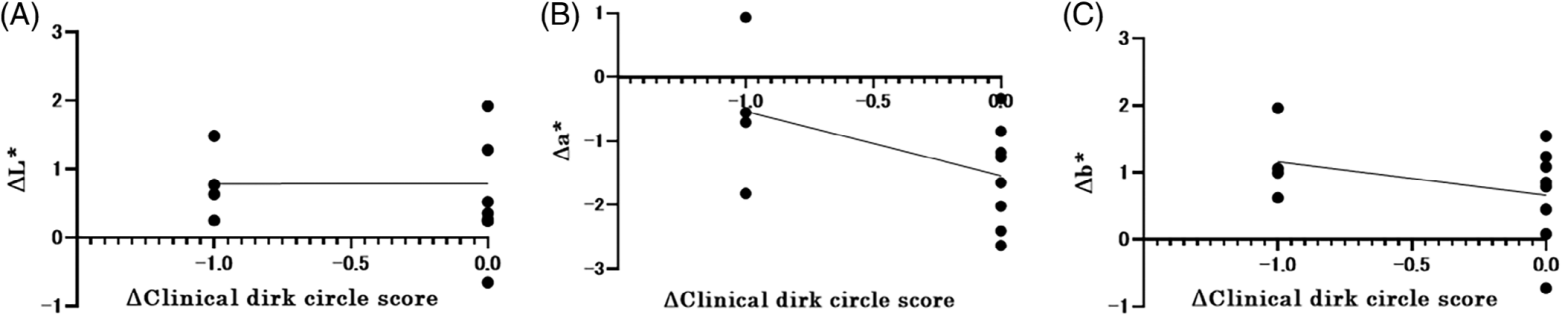
Correlation between change in score and change in spectrophotometric measurements in the subjects/group with mild periocular dark circles (baseline CDC score 0−1). (A) Correlation between change in CDC score and change in L*. No significant correlations are observed. (B) Correlation between change in CDC score and change in a*. A weak negative correlation is observed. (Spearman's rank correlation coefficient, *r* = −0.461, *p* = 0.154). (C) Correlation between change in CDC score and change in b*. No significant correlations were observed. CDC score: clinical dark circle score.

In many cases, the dark circles are caused by a complex interaction between several factors, including color tone and edema. Therefore, we measured the correlation between the amount of change in the CDC score and that in various parameters in each case and evaluated the stratification by the CDC score at baseline. We found a weak negative correlation between the change in CDC score and blood flow measured with a laser perfusion device (Spearman's rank correlation coefficient, *r* = −0.32, *p* = 0.0267). A similar weak negative correlation was also observed between the change in CDC score and TcPO_2_ (Spearman's rank correlation coefficient, *r* = −0.39, *p* = 0.027) (Figure 7A,B). The same analysis was performed for the subjects/group with mild periocular dark circles (0 or 1) and the subjects/group with severe periocular dark circles (2 or 3). A strong negative correlation was found between the change in CDC score and blood flow as measured by laser blood flowmetry in the subjects/group with mild periocular dark circles (Spearman's rank correlation coefficient, *r* = −0.615, *p* = 0.049). Similarly, a strong negative correlation was found between the change in CDC score and the change in TcPO_2_ (Spearman's rank correlation coefficient, *r* = −0.615, *p* = 0.049; Figure 8A–C). Furthermore, a strong negative correlation was observed between the amount of change in the CDC score and the amount of change in a* (Spearman's rank correlation coefficient, *r* = −0.46, *p* = 0.15; Figure 9A–C) in the subjects/group with mild periocular dark circles. In contrast, no correlations were found for any of the measures in the subjects/group with severe periocular dark circles (Supplementary files 2A–C, 3A–C). These results suggest that eyelid edema improved in some patients and that blood flow to the lower eyelid skin increased, which was significant in the subjects/group with mild periocular dark circles. No device‐related ophthalmic adverse events were observed in any patient after 1 month.

## 4 DISCUSSION

It has long been suggested that typing on a computer or playing computer games on visual display terminals reduces the frequency of blinking, thereby worsening periocular dark circles.^10^ Th increase in the use of smartphones and tablets is predicted to exacerbate this trend. The results of our study showed that the L* and a* values significantly improved because of changes in the physiological parameters of the skin before and after treatment, suggesting that the use of the device increased the brightness of the periocular dark circles and improved the redness or color change caused by lower eyelid edema. In addition, the a* values correlated with the dermatologist's CDC score at baseline, suggesting a relationship between CDC severity and skin redness. Matsui reported that L* values and TcPO_2_ correlated with the gross severity score of periocular dark circles in a group of Brazilian patients, which is similar to the results of our study.^11^ Hester et al. also reported that L* values correlated with the severity of periocular dark circles, and L* values are an important indicator in their evaluation.^12^ The fact that L* values improved before and after the use of the device in our study, indicates that the severity of periocular dark circles was indeed reduced. In conclusion, the a* and L* values are useful as objective evaluation indices for dark circles.

The results of the current study showed that there was a larger improvement in lower lid blood flow in the subjects/group with mild periocular dark circles. These results suggest that severe periocular dark circles are caused by a combination of factors, including pigmentation and anatomical problems other than edema, and that some local inflammation may have caused the decrease in surface temperature after device use. These results suggest that patients with severe periocular dark circles should receive comprehensive treatments from cosmetic specialists. However, for the subjects/group with mild periocular dark circles, the device s improved periocular dark circles, making it a viable home cosmetic device.

The main purpose of this device is to improve the color tone changes caused by edema and prevent the deterioration of the orbicularis oculus muscle by moving it. There are many age‐related changes in the human face, from the skeleton to the muscles and skin.^13^ In the past, it was thought that the orbicularis oculi muscle hypertrophies with age, but it has recently been suggested that this may not be true.^14^, ^15^ Anatomical evidence has shown that the masseter and facial muscles atrophy with age.^16^ Since the orbicularis oculi muscle is also a type of skeletal muscle, activation of these muscles with this device may help prevent atrophy.

The safety and efficacy of percutaneous electrical stimulation of the orbicularis oculus in a group of patients with retinitis pigmentosa has been reported.^17^ In this report, the safety of applying a 1‐mA current to the eye was demonstrated. In addition, in this study, various ophthalmologic examinations were performed before and 1 month after use, and it was confirmed that there were no changes.

The EMS device used in this study has been reported to be effective not only in muscle training but also in combination with aerobic exercise and rehabilitation in the elderly.^18^, ^19^ The results of our study demonstrate the efficacy and safety of EMS in the emerging field of periocular dark circles. Further development is recommended.

It should be noted that the number of participants in this study was limited and the study period was short; therefore, caution should be observed in generalizing our results. In addition, this study could not observe the duration of the effect of the device on improving periocular dark circles. The results of this study also suggest that the device may only be effective for dark circles caused by inadequate blood flow, as it mainly improved the brightness and redness of periocular dark circles. Further studies should be conducted on a larger scale and over a longer period to further investigate the mechanism and persistence of this therapeutic effect. Botulinum toxin treatment of the orbicularis oculi muscle is commonly used for lower eyelid wrinkles. This is done in the hope that paralysis of the orbicularis oculi muscle will improve the wrinkles. But this device is a muscle stimulator. In theory, this should not make the wrinkles worse because the large muscle movements that cause the wrinkles do not occur, but as this has not been clinically proven, more evidence is needed in the future.

## 5 CONCLUSION

Electrical muscle stimulation could improve periocular dark circles, especially in the subjects/group with mild periocular dark circles, whereas the effect was not evident in the subjects/group with severe periocular dark circles. The safety of the product was also confirmed, as there were no abnormalities in the ophthalmologist's examination after 1 month use. Future studies should be conducted on a larger scale and over a longer period of time to further investigate the mechanism and persistence of the therapeutic effect.

## AUTHOR CONTRIBUTIONS

Yukiko Yasui, Hiroshi Kato, Shuntaro Ogura, Masayo Kimura, Aki Kato, Yoshio Hirano, Hiroshi Morita, and Tsutomu Yasukawa performed the experiments. Hiroshi Kato, Aki Kato, and Soshi Takeda designed the study. Ako Kurachi and Soshi Takeda provided essential reagents and tools. Yukiko Yasui and Hiroshi Kato analyzed the data. Yukiko Yasui, Hiroshi Kato, and Akimichi Morita wrote the manuscript.

## CONFLICT OF INTEREST STATEMENT

Hiroshi Kato has received joint research funding and technical fee from MTG Inc.

## ETHICS STATEMENT

This study was conducted in accordance with the principles of the Declaration of Helsinki and was approved by the Nagoya City University Ethics Review Committee. The study volunteers were informed orally in advance of the purpose, methods, safety considerations, and risks of the experiment, and we obtained written consent from them (Nagoya City University Ethics Committee approval number: 46‐22‐0009).

## Supporting information

Supporting Information

## Data Availability

Data sharing not applicable to this article as no datasets were generated or analyzed during the current study.
