# Supplementary material for: Electrical stimulation of the lower eyelid orbicularis oculi muscle improves periocular dark circles
Source: Skin Res Technol. 2024 Apr 14;30(4):e13678. doi: 10.1111/srt.13678 (PMC11016813; doi:10.1111/srt.13678)

Supplemental file 1

Product Specification

・Product name ReFa EMS EYE（tentative）

・Manufacturer　MTG Co., Ltd., Nagoya, Japan

・EMS output

Frequency = 20 Hz、Pulse shape = Square wave、Pulse duration = 50 ms

Lv1 = 5.50V, 0.78mArms

Lv2 = 6.37V, 0.87mArms

Lv3 = 7.24V, 0.98mArms

Lv4 = 8.11V, 1.08mArms

Lv5 = 8.97V, 1.18mArms

Lv6 = 9.84V, 1.28mArms

Lv7 =10.71V, 1.38mArms

Lv8 = 11.58V, 1.48mArms

Lv9 = 12.45V, 1.58mArms

Lv10 = 13.32V, 1.68mArms

Lv11 = 14.19V, 1.78mArms

Lv12 = 15.05V, 1.88mArms

Lv13 = 15.92V, 1.98mArms

Lv14 = 16.79V, 2.08mArms

Lv15 = 17.66V, 2.18mArms

※Voltage crossing±10% Electrical resistance＝500Ω

・Power Supply (lithium ion rechargeable battery)

Rated voltage=3.7V

Rated input capacity=180mArms

Battery cycle count=500

・Size 85×54×20mm(controller)、35mm×17mm×5mm (electrode pad)

・Weight 100g

electrode pad


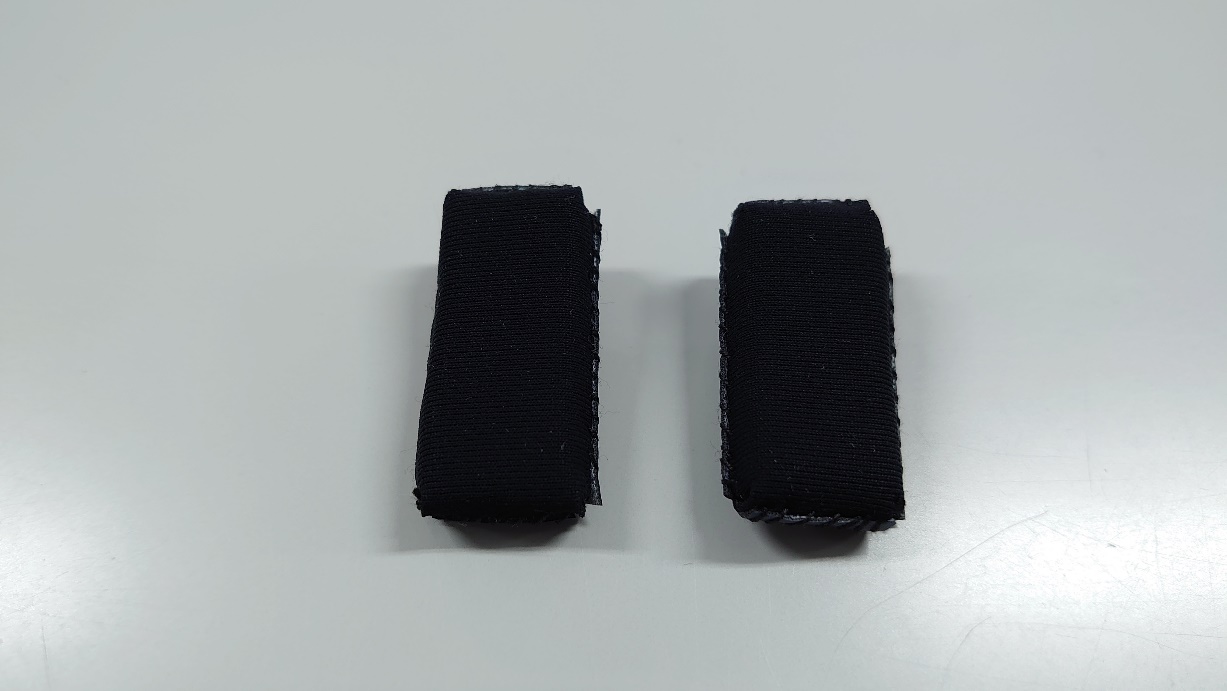

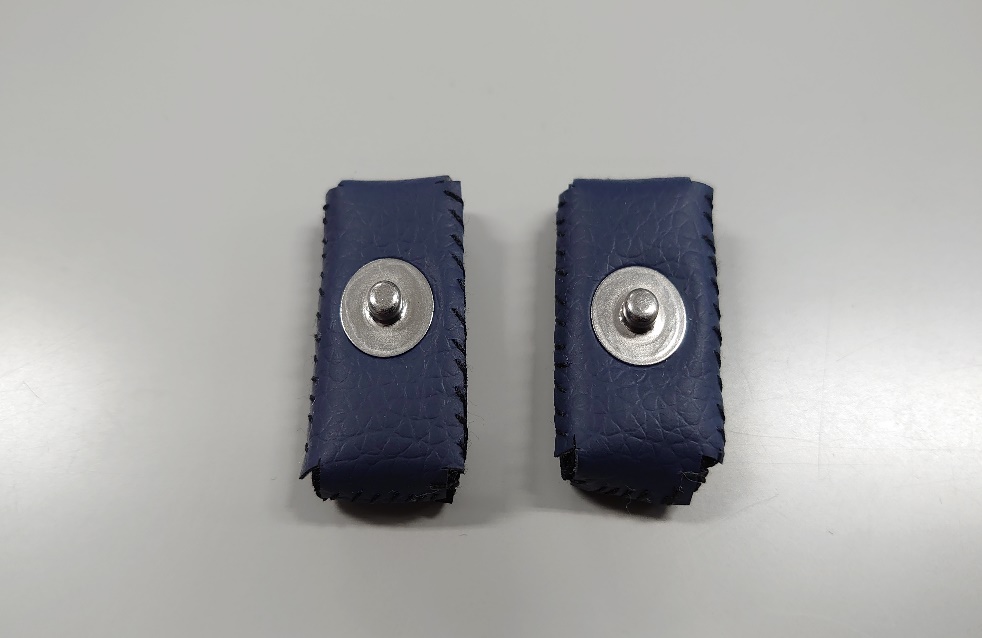


controller


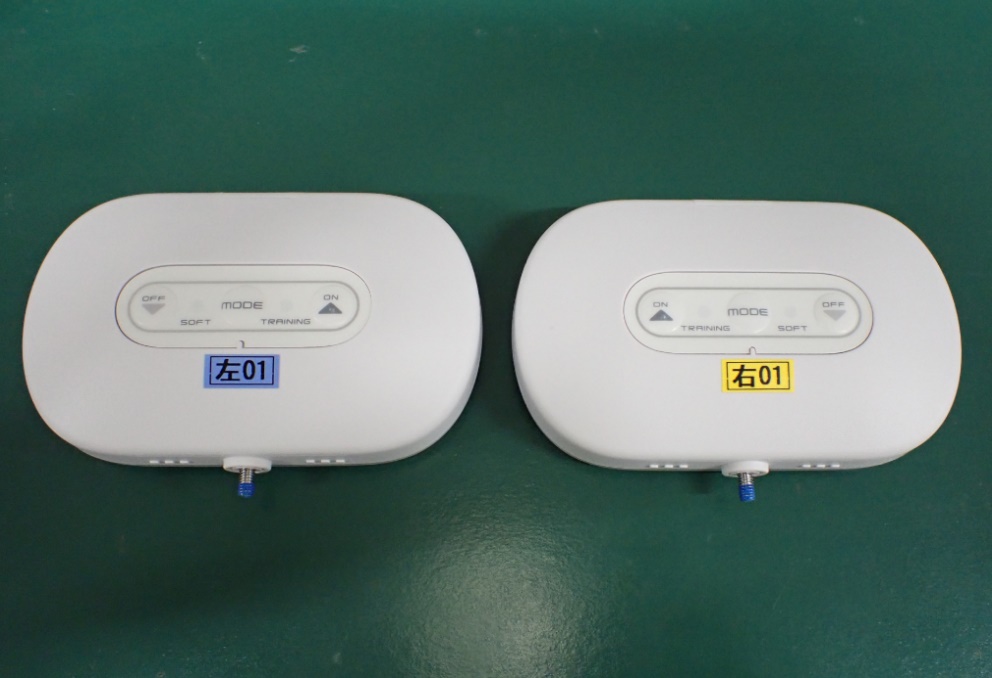


Supplemental file 2

a

b

c

Supplemental file 3


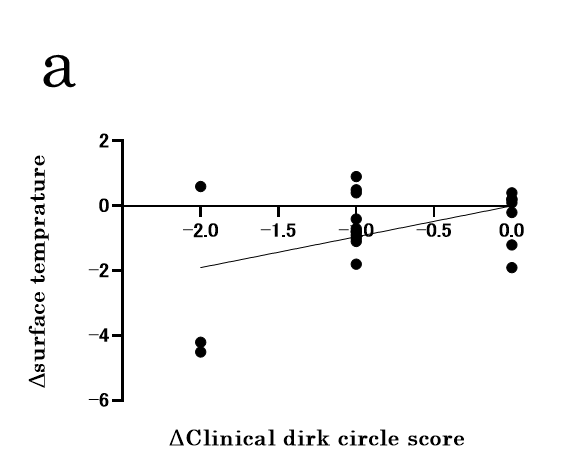

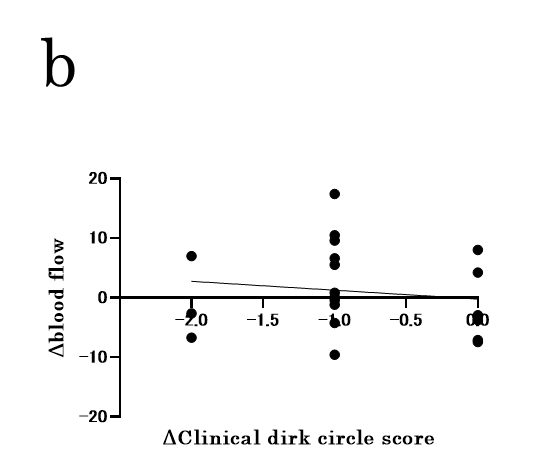


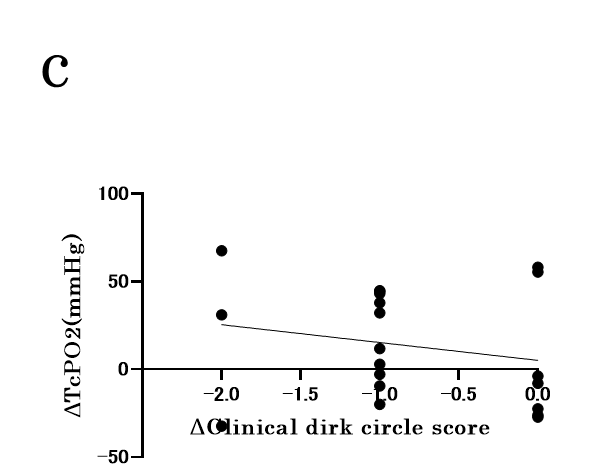

Supplement: Supplementary file 1 — Supporting Information [file SRT-30-e13678-s001.docx]
